# The German postgraduate degree program in ecotoxicology (SETAC GLB and GDCh): a success story

**DOI:** 10.1186/s12302-016-0078-5

**Published:** 2016-06-23

**Authors:** Klaus Peter Ebke, Jan Ahlers, Thomas Braunbeck, Jörg Oehlmann, Toni Ratte, Ralf B. Schäfer, Adolf Eisenträger, Andreas Schäffer

**Affiliations:** 1 Institute for Water Protection MESOCOSM GmbH, Neu-Ulrichstein 5, 35315 Homberg (Ohm), Germany; 2 Consultant [formerly Umweltbundesamt (Federal Environmental Agency)], Ahrenshooper Zeile 1A, 14129 Berlin, Germany; 3 Aquatic Ecology and Toxicology, Center for Organismal Studies, Heidelberg University, Im Neuenheimer Feld 230, 69120 Heidelberg, Germany; 4 Department Aquatic Ecotoxicology, Johann Wolfgang Goethe University Frankfurt am Main, Max-von-Laue-Str. 13, 60438 Frankfurt am Main, Germany; 5 ToxRat Solutions GmbH, Naheweg 15, 52477 Alsdorf, Germany; 6 Quantitative Landscape Ecology, Institute for Environmental Sciences, Fortstrasse 7, 76829 Landau, Germany; 7 Umweltbundesamt (Federal Environmental Agency), Wörlitzer Platz 1, 06844 Dessau-Rosslau, Germany; 8 Chair of Environmental Biology and Chemodynamics, RWTH Aachen, Worringerweg 1, 52056 Aachen, Germany

## Abstract

This article gives a comprehensive overview on the strategy, the development and the progress of the German postgraduate degree program in ecotoxicology (SETAC GLB and GDCh). The program soon prompted positive results: more than 10 years now the courses had an average enrolment rate of 90 %, and employment-seeking graduates from the first courses mostly succeeded in quickly finding employment relevant to their training. With over 450 students enrolled to date, the degree program contributes significantly to the field of Environmental Chemistry and Ecotoxicology.

**Electronic supplementary material:**

The online version of this article (doi:10.1186/s12302-016-0078-5) contains supplementary material, which is available to authorized users.

## Abstrakt

Der Artikel gibt einen umfassenden Überblick zu dem Postgradualstudiengang (PGS) Ökotoxikologie mit zertifiziertem Abschluss als Fachökotoxikologe (GDCh/SETAC GLB). Inzwischen kann auf eine 10-jährige Erfolgsgeschichte zurückgeblickt werden. Der Erfolg des Studiengangs stellte sich bereits sehr schnell ein: Die Kurse waren von Beginn an im Durchschnitt zu ca. 90 % ausgebucht, arbeitssuchende Absolventen der ersten Kurse fanden meist umgehend ausbildungsrelevante Anstellungen. Mit über 450 Teilnehmern leistet der Studiengang einen nennenswerten Beitrag in der Umweltchemie und der Ökotoxikologie.

**A German language Version of the article is provided as Additional file**1.

## Background

Following a suggestion by the German Federal Environmental Agency, a workshop on the situation of education and training in the field of ecotoxicology was held in October 2002. The event was prompted by the observation that training capacities and training institutions at universities could only insufficiently satisfy the demands for environmental chemists and ecotoxicologists in public authorities, enterprises, and research institutions. Representatives from science, industry, and public authorities adopted the “Berlin Manifesto on Ecotoxicology” which made suggestions on how to improve the training of young scientists and how to further develop ecotoxicology [3]. Some of these suggestions were promptly implemented. Particularly, the establishment of a postgraduate degree program [“Postgradualstudiengang” (PGS)] that certifies graduates as “Ecotoxicologists” provided a pragmatic solution for a timely implementation. Ideas on the required curriculum were discussed [2], and a respective curriculum was developed [4]. As early as 2005, the expert associations SETAC GLB (Society of Environmental Toxicology and Chemistry Europe, German-language branch e.V.) and GDCh (Gesellschaft Deutscher Chemiker e.V.), group ‘Environmental chemistry and ecotoxicology,’ signed a contract on the establishment of a new postgraduate degree program in ecotoxicology. Since then, both expert associations have held equal responsibility for the program. By now, the program looks back on a more then 10-year success story. The program soon prompted positive results: from the beginning, courses had an average enrolment rate of 90 %, and employment-seeking graduates from the first courses mostly succeeded in quickly finding employment relevant to their training. With over 450 students enrolled to date, the degree program contributes significantly to the field of environmental chemistry and ecotoxicology in Germany.

## The structure of the postgraduate degree program

**Fig. 1:**
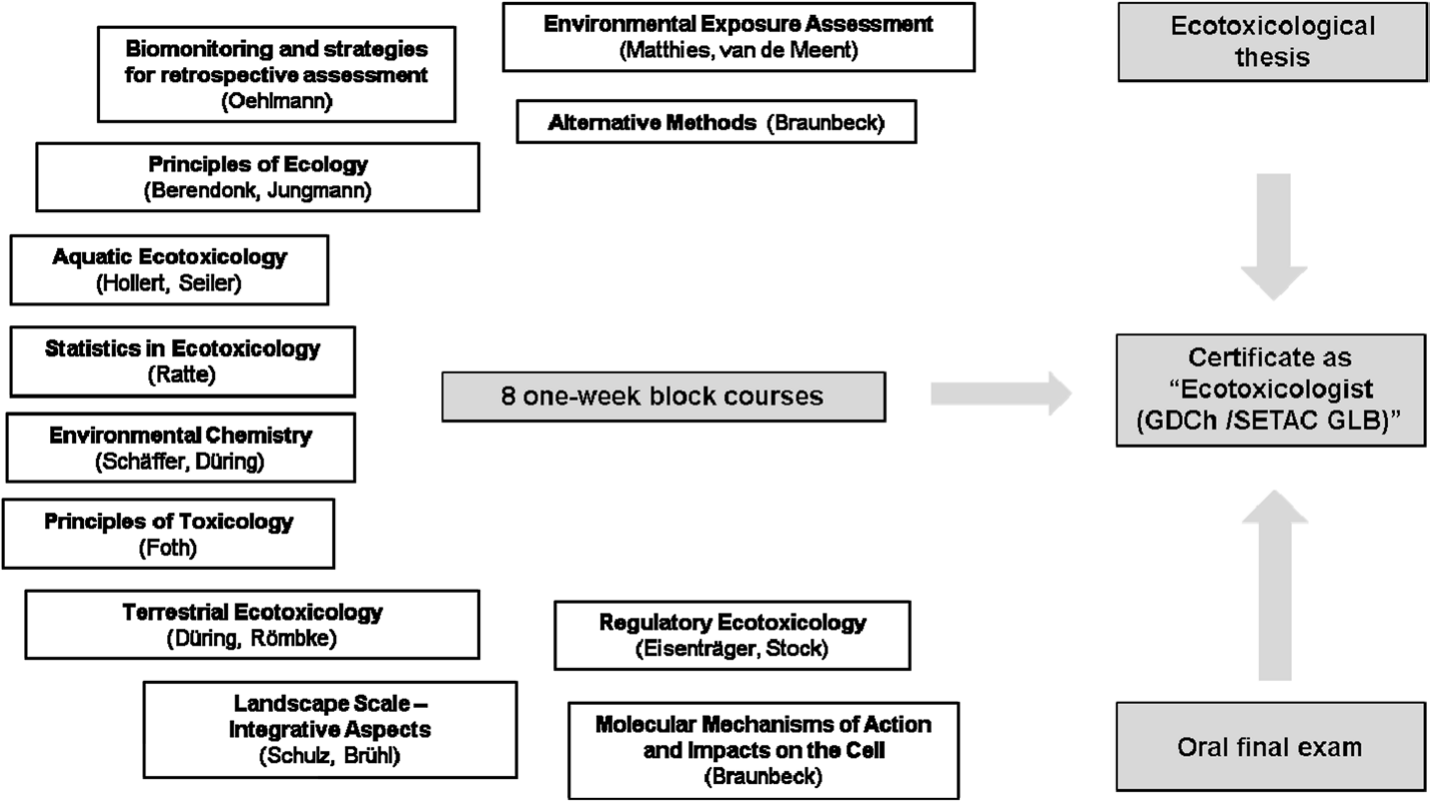
Composition of the postgraduate degree program with certificate as ecotoxicologist, including responsible persons for each of the thematic areas

In total 12 distinct courses (Fig. 1) with a duration of 5 days each are regularly offered at 11 different locations. To fulfill the requirements of the degree program, students have to participate in at least eight of the courses and pass the respective exams, write a scientific thesis and pass an oral final exam. The postgraduate degree program concludes with the certificate “Ecotoxicologist (GDCh/SETAC GLB).” Theses of particular interest are presented at the annual meetings of the expert associations involved.

The training addresses a broad range of topics and has been evolving continuously over the last 10 years. Since 2012 for example, Prof. Dr. M. Matthies at Osnabrück University teaches the course “Environmental exposure assessment,” and as of 2015 a new course on “Alternative methods” by Prof. Dr. T. Braunbeck at Heidelberg University is in preparation.

## Thematic areas

The course “Biomonitoring and strategies for retrospective assessment” centers on the use of organisms for the observation of the environment with special emphasis on the evaluation of the impact of already introduced chemicals on ecosystems. There are numerous cases of environmental chemicals in which biomonitoring revealed an environmental risk which had originally been underestimated and eventually led to an ex-post regulation of the substances. The course aims at providing an overview of the possibilities and limitations of biomonitoring in different environmental media (water with sediments, soil, atmospheric deposition) and at different biological integration levels (ranging from molecular markers to organisms and communities). In lectures, the tasks, strategies, principals, and types of bioindication are introduced together with the possibilities of chemical monitoring with a special focus on environmental specimen banks. Furthermore, the course discusses accumulation and effect monitoring using plants, invertebrates, fish, other vertebrates, and whole communities in aquatic and terrestrial systems. The use of biomarkers in biomonitoring as well as assessment models is also addressed. Additionally to the lectures, examples for biomonitoring techniques and methods are demonstrated in short laboratory courses.

The course “Landscape scale—integrative aspects” focuses on the assessment of effects of chemicals in non-target ecosystems. As the ecological complexity—such as additional stress through climatic influences or mixture effects of chemicals—is reduced in laboratory tests and controlled semi-field experiments, prediction of field effects is subject to uncertainty. In addition, when observing chemical exposure and effects on the landscape scale, spatial aspects need to be considered, for example possible recovery mechanisms through adjacent populations. Overall, the course provides the principals of ecotoxicology on the complex landscape scale.

Topics in the “Environmental chemistry” course include the composition and properties of the environmental media soil, water, and air with regard to the interaction with environmental pollutants; the basics of chemical heavy metal analytics, including the analysis of metal species; analysis of organic pollutants and biomimetic tools; physicochemical properties of environmental chemicals as well as their transformation reactions and the assessment of their biological degradability. Relevant publications of these research fields are presented by the participants and discussed. A demonstration lab tour introduces the participants to important environmental analytical methods.

The basics of “Regulatory ecotoxicology” [1] are treated in a course at the Umweltbundesamt (Federal Environmental Agency). Legal and functional/technical aspects of the assessment and the management of pesticides, biocides, medicinal products for human and animal use as well as chemicals (REACH) are demonstrated and discussed. To provide a basis for this, the foundations of hazard- and risk-based assessment of chemicals and the resulting consequences for the authorization and restriction management are introduced.

The course “Molecular mechanisms of action and impacts on the cell” provides an overview of the following main topics: (1) structure and function of the cell and impairment of cellular processes (e.g., Ca^2+^ homeostasis, oxidative phosphorylation, enzyme inhibition, oxidative stress, biotransformation), (2) effects on cellular structures (membranes, organelles, nucleus), (3) imaging techniques: histology, fluorescence-, electron- and light microscopy; confocal laser scanning microscopy, (4) effects on the cell: cytotoxicity, genotoxicity, mutagenicity, carcinogenicity; detoxification, repair and protection mechanisms (e.g., cytochrome P450, metallothionein, stress proteins), (5) cell culture and cytotoxicity tests (different endpoints), (6) specific effects: biochemistry, biotransformation, Hsp70, (7) genotoxicity and mutagenicity (comet assay and Ames test), (8) genomics, proteomics, and microarrays. The theoretical presentations are complemented by various demonstrations in the laboratory.

The course “Terrestrial ecotoxicology” enlarges upon the following subjects: (1) introduction to terrestrial ecotoxicology, especially the distinction between prospective and retrospective risk assessment in soils, (2) estimating and testing the behavior of chemicals in soils, with a particular focus on the exposition of organisms, (3) soils and their biological properties, with special focus on biodiversity, and ecological functions, (4) overview of terrestrial testing systems with microorganisms, plants and invertebrates at laboratory, semi-field and field level, (5) presentation of methods to assess the effects of pesticides on bees and non-target arthropods, (6) the use of birds in terrestrial ecotoxicology (particularly in the risk assessment of pesticides), (7) regulatory approaches for the risk assessment of pesticides, in particular for non-target and soil organisms, (8) testing and evaluating veterinary pharmaceuticals. In the context of the course, individual tests are either demonstrated or, if possible, executed by the participants themselves, in particular earthworms and small mammals. Finally, the research center Neu-Ulrichstein can be visited.

The course “Principles of toxicology” offers an overview of the essential principals of (human) toxicology. The basic problems of a harmful effect of chemicals at the workplace, in the private sphere or the environment, are illustrated by well-documented examples (metals, polycyclic carbohydrates, pesticides). The essential concepts of toxicology are explained, elaborated by reference to the examples and presented in their relation to mechanisms of action, adaptations and reversible/irreversible dysfunctions. The course is designed to provide a basis for subsequent courses. At the end, the participants should be able to understand relevant terms in their meaning and should have obtained a general view of strategies of toxicological assessment. It is not intended to give a taut presentation of the full discipline of toxicology but rather to make basic principles and distinct features of human toxicology comprehensible.

The course “Aquatic ecotoxicology” gives an introduction into the basic terms and definitions of (aquatic) ecotoxicology: (1) ecotoxicological standard tests I: freshwater algae and cyanobacteria growth inhibition test, *Lemna* sp. growth inhibition test (OECD 201, OECD 221), *Daphnia* sp. Acute immobilization test, Daphnia magna reproduction test (OECD 202, OECD 211) as well as the sediment–water chironomid toxicity test (OECD 218/219), for the prospective assessment of effects of substances in water and sediment. (2) Good laboratory practice (GLP), requirements for authorization-relevant testing procedures; preparation and evaluation of algae and acute Daphnia tests in compliance with GLP, (3) ecotoxicological standard tests II: toxicity tests with fish; fish embryo testing as an animal alternative method, (4) demonstrations of fish/fish embryo assays, (5) ecotoxicological testing at higher levels I: testing on populations (basics of population dynamics; preparation, implementation, evaluation), (6) ecotoxicological tests at higher levels II: aquatic mesocosm studies (background; preparation, implementation, sampling), (7) ecotoxicological tests at higher levels III: experiments with lotic ecosystems (gutter systems) (background, implementation, endpoints, evaluation), (8) marine ecotoxicology, (9) toxicokinetics and bioaccumulation.

The course “Principles of ecology” imparts fundamental relationships in ecology. It presents habitats and their environmental factors, proceeding from the level of the individual to populations and outlines interactions within ecosystems. In addition to the classical methods in ecology, modern methods of analyzing the structure and variability of populations are introduced. The main focus of the course is on the limnetic habitat. Aside from marine ecology the forest ecosystem, particularly tropical forests and forest soil, is presented, too. Furthermore, competition experiments, and the determination and modeling of the population dynamics of Daphnia are part of the practical exercises.

The course “Statistics in ecotoxicology” treats the statistical analysis of single-species tests. It aims at imparting the essential basic principles of statistical testing in general and at enabling the participants to choose appropriate statistical tests, perform them technically correct and evaluate their results with expert knowledge. A particular focus is put on the scaling of data, because the choice of the appropriate test decisively depends on the underlying data scale. Basic terms such as ‘sample distribution,’ ‘one-sided/-tailed vs. two-sided/-tailed testing,’ ‘significance level,’ etc. are clarified and typical statistical parameters defined. Based on this, a general framework for choosing and performing statistical tests is presented and different tests for the evaluation of biotests are discussed [pretests for outliers, normal distribution and variance homogeneity, comparisons with standards, statistical testing for significant differences in the effects shown by different testing approaches (e.g., limit-test, tests against solvent controls), general proof of an effect (analysis of variance, ANOVA), verification of threshold concentrations through multiple tests (NOEC/LOEC), Bonferroni correction, ß-errors and power of statistical tests, minimum detectable difference (MDD)].

Another main topic is the modeling of data (e.g., dose–response-functions). The possibility of adapting functions is addressed, presenting and discussing common models for dose–response functions (e.g., probit, Weibull and logit models; dose–response-functions with more than two parameters) and methods for adapting them (linear and non-linear regression). The concepts of ECx and NOEC are compared and their respective advantages and disadvantages explained. The theoretical part is illustrated and consolidated by way of practical exercises.

The course “Environmental exposure assessment” was included in the curriculum since 2015 after a 2-year pilot phase, which was supported by the Fonds der Chemischen Industrie (FCI). The students learn the basics for mathematical modeling of transport and transformation of chemicals in the environment. Based on mass balancing in one or several compartments, the concepts for calculating concentrations in air, soil, water, and sediment are compiled and transformed into mathematical equations before students independently deepen their knowledge with exercises. Different levels of complexity are treated, ranging from the partition equilibrium to dynamic behavior as well as uptake by plants and animals and transfer in food chains. The influence of physical–chemical substance properties and environmental conditions on the concentration in the various environmental media are examined. In case studies, the exposure (PEC = Predicted environmental concentration) for individual chemicals is estimated exemplarily and compared with corresponding (eco-)toxicity data (PNEC = Predicted no effect concentration) to assess the risk for humans and ecosystems. In group work, approaches for refining the estimation of exposure are discussed and the results presented to all participants. The software necessary for calculations is provided for the participants. The course is held in English language.

New: “Alternative methods” started in 2015. The course covers the following topics: (1) ethical foundations for the protection of animals as fellow creatures, (2) legal basis of animal protection, (3) statistics of animal testing, (4) examples for alternatives to acute toxicity tests with fish (e.g., cell culture assays, fish embryo tests), (5) specific endpoints in alternative methods on cell cultures and fish, (6) basic principles in the development and validation of alternative testing methods (e.g., ECVAM approach, OECD guidelines), (7) integration of alternative methods in intelligent testing strategies (e.g., OECD fish testing strategy), (8) non-testing strategies (e.g., waving, read across, QSAR-strategies), (9) alternative approaches for determining bioaccumulation, (10) screening with lower vertebrates as an alternative to teratogenicity tests with vertebrates, (11) toxicogenomics as an alternative to conventional testing methods. The modules ‘cell culture’ and ‘fish embryo testing’ are complemented by corresponding demonstrations in the laboratory.

## Organization

The PGS panel comprises six experts each one dispatched by SETAC GLB and GDCh, and four selected by the course instructors (for current members, see Table 1). The PGS panel selects a chairperson and a deputy. The panel decides on all issues of interest for the degree program, e.g., sets up the examination regulations and appoints course instructors. The administrative office of the SETAC GLB at the Research Centre Neu-Ulrichstein, Homberg (Ohm) is responsible for the administration of the postgraduate degree program and is, amongst other issues, responsible for accounting, examination office, coordination, finances, and administration of the website.

**Table 1:** Roles and responsibilities in the postgraduate degree program ecotoxicology

PGS panel	2014–2015	
Chairperson	Jun.-Prof. Dr. Ralf B. Schäfer	Institute for Environmental Sciences, University of Koblenz-Landau
Deputy chairperson	Prof. Dr.-Ing. Adolf Eisenträger Dr. F. Stock	Umweltbundesamt (Federal Environmental Agency) Dessau-Rosslau
Member	Prof. Dr. Thomas Braunbeck	Institute for Zoology, Heidelberg University
Member	Prof. Dr. Jörg Oehlmann	Department Aquatic Ecotoxicology, Johann Wolfgang Goethe University Frankfurt
Member	Prof. Dr. Andreas Schäffer	Chair of Environmental Biology and Chemodynamics, RWTH Aachen
Member	Dr. Andreas Willing	BASF Personal Care and Nutrition GmbH, Düsseldorf
*Organization*	SETAC GLB—administrative office	Mesocosm GmbH at FNU Research Centre Neu-Ulrichstein, Homberg (Ohm)
Managing director	Prof. Dr. Klaus Peter Ebke	Institute for Water Protection MESOCOSM GmbH, Homberg (Ohm)

## Performance figures of the postgraduate degree program

Since 2004, 60 courses have been held with a total of 1300 participants. About 34 % of the participants were from industries, 14 % from small- and medium-sized enterprises (SME), 25 % from public authorities, 19 % were students, and 9 % were employment seekers.

A total of 450 persons have been registered at the examination office of the degree program, and in 2015 there are 315 active participants 49 % of which have already passed more than four courses.

Out of a total of 60 courses offered, only four had to be canceled due to a lack of participants.

The PGS panel is proud that 24 graduates have already successfully accomplished the degree program. It took participants about 2–4 years to fully complete the program.

## Interim result

Currently, most of the courses of the postgraduate degree program are fully booked. Thus, early registration is mandatory to be able to participate.

The second issue requested by the Berlin Manifesto, the establishment of M.Sc. programs in ecotoxicology, has meanwhile been realized at the Universitiesy of Koblenz-Landau, Duisburg-Essen and Aachen. Moreover, more than 20 other German universities offer thematic programs or individual courses on ecotoxicology as part of various degree programs. Also in this context, the postgraduate degree program “Ecotoxicologist (GDCh/SETAC GLB)” has provided important stimuli.

## Additional file

10.1186/s12302-016-0078-5 The German version of original article.
