# Supplementary material for: The German postgraduate degree program in ecotoxicology (SETAC GLB and GDCh): a success story
Source: Environ Sci Eur. 2016 Jun 23;28(1):19. doi: 10.1186/s12302-016-0078-5 (PMC5044969; doi:10.1186/s12302-016-0078-5)
Supplement: Supplementary file 1 — 10.1186/s12302-016-0078-5 The German version of original article. [file 12302_2016_78_MOESM1_ESM.doc]

**Beitragsserie Fachökotoxikologinnen/en (GDCh/SETAC GLB)**

**Fachökotoxikologinnen/en (GDCh/SETAC GLB) - eine Erfolgsgeschichte des Postgradualstudiengangs Ökotoxikologie**

Klaus Peter Ebke^1*^, Jan Ahlers^2^, Thomas Braunbeck^3^, Jörg Oehlmann^4^, Toni Ratte^5^, Ralf B. Schäfer^6^, Adolf Eisenträger^7^, Andreas Schäffer^8^

^1^ Institut für Gewässerschutz MESOCOSM GmbH, Neu-Ulrichstein 5, D-35315 Homberg (Ohm)

^2^ Consultant (ehemals Umweltbundesamt), Ahrenshooper Zeile 1A, D-14129 Berlin

^3^ Aquatische Ökologie & Toxikologie, Center for Organismal Studies, Universität Heidelberg, Im Neuenheimer Feld 230, D-69120 Heidelberg

^4^ Department Aquatic Ecotoxicology, Johann Wolfgang Goethe University Frankfurt am Main, Max-von-Laue-Str. 13, D-60438 Frankfurt am Main

^5^ ToxRat Solutions GmbH, Naheweg15, D-52477 Alsdorf

^6^ Quantitative Landschaftsökologie, Institut für Umweltwissenschaften, Fortstrasse 7, D-76829 Landau

^7^ Umweltbundesamt (Federal Environmental Agency), Wörlitzer Platz 1, D-06844 Dessau-Rosslau

^8^ Lehrstuhl für Umweltbiologie und -chemodynamik, RWTH Aachen, Worringerweg 1, D-52056 Aachen

* Korrespondenzautor: ebke@mesocosm.de, Tel. 06633-642740

*Abstract*

Dieser Artikel gibt einen Überblick über das Konzept der Ausbildung zum Fachökotoxikologen (GDCh/SETAC GLB), die Ausbildungsinhalte werden vorgestellt und die Entwicklung des Studiengangs wird beschrieben. Das postgraduale Ausbildungsprojekt startete in 2004 und blickt heute auf eine mehr als 10-jährige Erfolgsgeschichte im über 600 durchgeführten Kursen zurück. Mit einem Teilnehmerspektrum aus Behörden, Industrie und den Hochschulen und 12 unterschiedlichen Themen-Schwerpunkten hat diese Ausbildung deutlichen Einfluss auf den Bereich der Umweltchemie und Ökotoxikologie genommen.

*Einleitung*

Im Oktober 2002 fand auf Anregung des Umweltbundesamtes ein Workshop zur Ausbildungssituation in der Ökotoxikologie statt. Anlass war die Beobachtung, dass der Bedarf an Umweltchemikern und Ökotoxikologen in Behörden, Unternehmen und Forschungseinrichtungen durch die Ausbildungskapazitäten und die Ausbildungsausrichtungen an den Universitäten nicht ausreichend gedeckt werden kann. Vertreter aus Wissenschaft, Industrie und Behörden verabschiedeten das „Berliner Manifest Ökotoxikologie“, in dem Vorschläge zur Verbesserung der Ausbildung des wissenschaftlichen Nachwuchses und der wissenschaftlichen Weiterentwicklung der Ökotoxikologie unterbreitet wurden (Ahlers et al. 2003). Einige dieser Vorschläge wurden schnell aufgegriffen. Insbesondere die Etablierung eines Postgradualstudiums mit zertifiziertem Abschluss als Fachökotoxikologe erschien als pragmatische Lösung für eine zeitnahe Umsetzung. Es wurden Vorstellungen zu den erforderlichen Lehrinhalten diskutiert (Ahlers et al. 2004) und ein entsprechendes Curriculum entwickelt (Hollert et al. 2005). Bereits 2005 schlossen die beiden Fachgesellschaften SETAC GLB (Society of Environmental Toxicology & Chemistry Europe, German-Language Branch e.V.) und GDCh (Gesellschaft Deutscher Chemiker e.V.) Fachgruppe 'Umweltchemie und Ökotoxikologie' einen Vertrag zur Gründung eines neuen Postgradualstudiengangs (PGS) Ökotoxikologie. Die beiden Fachgesellschaften tragen gleichberechtigt die Verantwortung für den Studiengang. Inzwischen kann auf eine über 10-jährige Erfolgsgeschichte zurückgeblickt werden. Der Erfolg des Studiengangs stellte sich bereits sehr schnell ein: Die Kurse waren von Beginn an im Durchschnitt zu ca. 90 % ausgebucht, arbeitssuchende Absolventen der ersten Kurse fanden meist umgehend ausbildungsrelevante Anstellungen. Mit über 450 Teilnehmern leistet der Studiengang einen nennenswerten Beitrag in der Umweltchemie und der Ökotoxikologie.

*Die Struktur des Postgradualstudiengangs*

Insgesamt werden 12 verschiedene Kurse (Abb. 1) mit einer Dauer von jeweils 5 Tagen an 10 verschiedenen Standorten angeboten. Für einen erfolgreichen Abschluß des Curriculums müssen 8 der angebotenen Kurse besucht und mit einer Prüfung abgeschlossen werden, eine wissenschaftliche Arbeit muss angefertigt und eine mündliche Abschlussprüfung absolviert werden. Das PGS wird mit dem Zertifikat „Fachökotoxikologin / Fachökotoxikologe (GDCh / SETAC GLB)“ abgeschlossen. Besonders interessante Abschlussarbeiten werden auf den Jahrestagungen der beteiligten Fachgesellschaften vorgestellt.

**
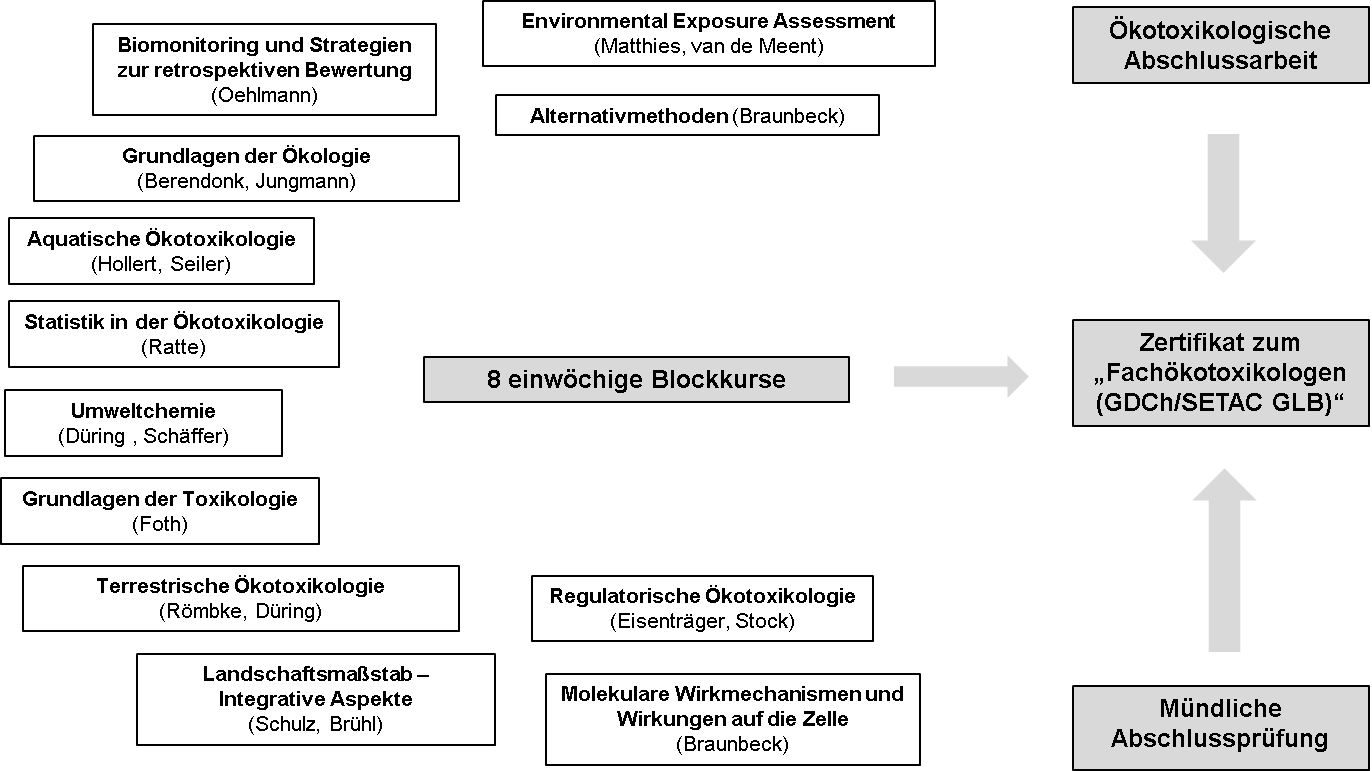
**

**Abb.1:** Konzeption des Postgradualstudiums mit zertifiziertem Abschluss als Fachökotoxikologe und die Verantwortlichen für die einzelnen Themenblöcke.

Die Ausbildung zeichnet sich durch eine breite Themenbasis aus und ist in den letzten Jahren weiter gewachsen. So wird seit 2012 der Kurs „Environmental Exposure Assessment“ von Prof. Dr. M. Matthies an der Universität Osnabrück angeboten, und für 2015 ist ein neuer Kurs „Alternative Methoden“ von Prof. Dr. T. Braunbeck an der Universität Heidelberg in Vorbereitung.

*Die Themengebiete*

Im Kurs "**Biomonitoring und Strategien zur retrospektiven Bewertung**" steht die Umweltüberwachung unter Einsatz von Organismen im Vordergrund. Dabei spielt die ökotoxikologische Bewertung von bereits eingeführten Substanzen eine wesentliche Rolle. So gibt es zahlreiche Beispiele von Umweltchemikalien, für die sich über das Biomonitoring Hinweise auf eine anfänglich unterschätze Umweltgefährdung ergaben, die letztlich zu einer nachträglichen Regulierung der Substanzen führte. Ziel des Kurses ist es, eine Übersicht zu den Möglichkeiten und Grenzen des Biomonitorings in den verschiedenen Umweltmedien (Wasser mit Sedimenten, Boden, atmosphärische Deposition) und auf unterschiedlichen biologischen Integrationsebenen (molekulare Marker über Organismen bis zu Lebensgemeinschaften) zu geben. Im Kurs werden in Vorträgen die Aufgaben, Strategien, Prinzipien und Typen von Bioindikation und Biomonitoring ebenso vorgestellt, wie die Möglichkeiten des chemischen Monitorings unter besonderer Berücksichtigung von Umweltprobenbanken. Weiterhin wird auf das Akkumulations- und Effektmonitoring mit Pflanzen, Wirbellosen, Fischen, anderen Wirbeltieren und kompletten Lebensgemeinschaften in aquatischen und terrestrischen Systemen eingegangen. Auch der Einsatz von Biomarkern im Biomonitoring sowie Bewertungsmodelle werden thematisiert.

Der Kurs „**Landschaftsmaßstab – Integrative Aspekte**“ befasst sich mit der Beurteilung der Auswirkung von Chemikalien in Nichtzielökosystemen. Da bei Laborversuchen und kontrollierten Freilandexperimenten die ökologische Komplexität, beispielsweise zusätzlicher Stress durch klimatische Einflüsse oder Kombinationswirkungen von Chemikalien, reduziert ist, sind die Auswirkungen im Freiland nicht immer eindeutig vorhersagbar. Zudem kommen bei der Betrachtung von Chemikalienexposition und -wirkungen im Landschaftsmaßstab auch räumliche Aspekte hinzu wie zum Beispiel mögliche Kompensationsmechanismen durch benachbarte Populationen. Insgesamt vermittelt der Kurs die wesentlichen Prinzipien der Ökotoxikologie im komplexen Landschaftsmaßstab.

Die „**Umweltchemie**“ behandelt die Zusammensetzung und die Eigenschaften der Umweltmedien Boden, Wasser und Luft in Bezug auf die Wechselwirkung mit Umweltschadstoffen und deren Verbleib, Grundlagen der chemischen Analytik von Schwermetallen inklusive Speziesanalytik und von Organika, den Einsatz von Biomimetika für die zeitintegrierte Analyse von Schadstoffen, physikochemische Eigenschaften von Umweltchemikalien sowie deren abiotischen und biologischen Transformationsreaktionen. Anhand von Vorlesungen und der Besprechung aktueller Veröffentlichungen werden diese Themen weiter vertieft. Ein Demonstrationspraktikum führt die Teilnehmer in wichtige umweltanalytische Anwendungen ein.

Die Grundlagen der „**Regulatorischen Ökotoxikologie**“ (Ahlers et al.2008) werden in einem Kurs im Umweltbundesamt behandelt. Die gesetzlichen und fachlichen Aspekte der Bewertung und des Managements von Pflanzenschutzmitteln, Bioziden, Human- und Tierarzneimitteln sowie Chemikalien (REACH) werden aufgezeigt und diskutiert. Hierzu werden eingangs die Grundlagen der gefährlichkeits- und der risikobasierten Bewertung von Chemikalien vermittelt und die daraus resultierenden Konsequenzen für das Management (Zulassung und Beschränkung) aufgezeigt.

Der Kurs „**Molekulare Wirkmechanismen und Wirkungen auf die Zelle**“ gibt einen Überblick über folgende Themenschwerpunkte: (1) Struktur und Funktion der Zelle und Beeinträchtigung zellulärer Prozesse (z.B. Ca2+-Homöostase, oxidative Phosphorylierung, Enzymhemmung, oxidativer Stress, Biotransformation), (2) Wirkungen auf zelluläre Strukturen (Membranen, Organellen, Zellkern), (3) bildgebende Verfahren: Histologie, Fluoreszenz-, Elektronen- und Lichtmikroskopie, konfokales Laser-Scanning-Mikroskop, (4) Wirkungen auf die Zelle: Zytotoxizität, Gentoxizität, Mutagenität, Kanzerogenese, Entgiftungs-, Reparatur- und Schutzprozesse (z.B. Cytochrom P450, Metallothioneine, Stressproteine), (5) Zellkultur und Zytotoxizitätstests (verschiedene Endpunkte), (6) spezifische Wirkungen I: Biochemie, Biotransformation, HSP70, (7) spezifische Wirkungen II: Gentoxizität und Mutagenität (Comet-Assay und Ames-Test), (8) Genomics, Proteomics, Microarrays. Die theoretischen Darstellungen werden durch diverse Demonstrationen im Labor ergänzt.

Der Kurs „**Terrestrische Ökotoxikologie**“ vertieft folgende Themen: (1) Böden und ihre pedologischen und biologischen Eigenschaften mit besonderer Berücksichtigung der Biodiversität, Funktionen und Leistungen der Bodenbiozönosen, (2) gesetzliche Anforderungen zur Chemikalienregistrierung (speziell Pestiziden) und zur Beurteilung kontaminierter Böden, (3) Abschätzung bzw. Testung des Verhaltens von Chemikalien im Boden unter besonderer Berücksichtigung der Exposition von Organismen, (4) Überblick über terrestrische Testsysteme mit Mikroorganismen, Pflanzen und Invertebraten auf der Labor-, Halbfreiland- und Freilandebene, (5) Einsatz von Wirbeltieren (Vögel, Kleinsäuger) und Nicht-Ziel-Arthropoden in der terrestrischen Ökotoxikologie (speziell bei der Risikobeurteilung von Pestiziden), (6) Nutzung terrestrischer Tests zur prospektiven und retrospektiven Bewertung in Böden, (7) die Verwendung aquatischer Tests (inklusive solchen zur Genotoxizität) bei der Beurteilung der Rückhaltefunktion von Böden, (8) Testung und Beurteilung von Veterinärpharmaka. Zudem werden im Rahmen des Kurses einzelne Testverfahren bzw. Testorganismen sowohl im Labor (z.B. Bodenalgen, Nematoden) als auch im Freiland (z.B. der Fang von Regenwürmer) entweder demonstriert oder, soweit möglich, von den Teilnehmern selbst durchgeführt.

Der Kurs „**Grundlagen der Toxikologie**“ bietet einen Überblick über die wesentlichen Prinzipien der (Human-) Toxikologie. Die grundlegenden Probleme einer Schadwirkung durch Chemikalien am Arbeitsplatz, im privaten Umfeld oder in der Umwelt sollen anhand gut dokumentierter Beispiele illustriert werden (Metalle, polycyclische Kohlenwasserstoffe, Pestizide). Es sollen die wesentlichen Begriffe der Toxikologie erklärt, anhand der Beispiele ausgeführt und in ihrem Kontext zu Wirkmechanismen, Anpassungen und reversiblen/irreversiblen Dysfunktionen präsentiert werden. Der Kurs soll u.a. die Grundlage für weiterführende Kurse legen. Die Teilnehmer sollen am Ende wichtige Begriffe in ihrer Bedeutung einordnen können und eine Übersicht über Strategien der toxikologischen Beurteilung haben. Es ist nicht angestrebt, die Toxikologie in ihrer Breite komprimiert darzustellen, sondern es sollen grundlegende Prinzipien und Besonderheiten in der Humantoxikologie verstehbar werden.

Der Kurs „**Aquatische Ökotoxikologie**“ gibt eine Einführung in Grundbegriffe und Definitionen der (aquatischen) Ökotoxikologie: (1) ökotoxikologische Standardtests I : Biotests mit Algen und Lemnaceen (OECD 201, OECD 221), akuter und chronischer Daphnientest (OECD 202, OECD 211) sowie Chironomustest (OECD 218/219) zur prospektiven Bewertung von Substanzeffekten in Wasser und Sediment, (2) GLP Gute Labor Praxis, Anforderungen an zulassungsrelevante Prüfungen; Ansetzen und Auswertung von Algen- und akutem Daphnientest nach GLP, (3) ökotoxikologische Standardtests II: Tests mit Fischen; Fischembryotest als Ersatzmethode, (4) Demonstrationen zum Fisch-/Fischembryotest, (5) ökotoxikologische Tests auf höheren Stufen I: Tests mit Populationen (Grundlagen der Populationsdynamik; Ansetzen; Durchführung, Auswertung), (6) ökotoxikologische Tests auf höheren Stufen II: Aquatische Mesokosmosversuche (Background; Ansetzen Durchführung; Probenahme), (7) ökotoxikologische Tests auf höheren Stufen III: Versuche mit Fließrinnen (Background, Durchführung, Endpunkte, Auswertung), (8) marine Ökotoxikologie, (9) Toxikokinetik und Bioakkumulation.

Der Kurs „**Grundlagen der Ökologie**“ gibt einen Einblick in wichtige Zusammenhänge in die Ökologie. Ziel ist es, den Lebensraum mit den Umweltfaktoren darzustellen und, über die Ebene des Individuums hinausgehend, Populationen und schließlich Wechselwirkungen innerhalb von Ökosystemen zu erläutern. Neben klassischen Methoden in der Ökologie werden auch moderne Methoden der Analyse von Struktur und der Variabilität von Populationen vermittel. Der limnische Lebensraum ist Schwerpunkt des Kurses, neben mariner Ökologie wird auch das Ökosystem Wald speziell der Tropenwald und Waldböden vorgestellt. Weiterhin sind Konkurrenzexperimente, Bestimmung der Populationsdynamik von Daphnien und deren Modellierung Bestandteil der praktischen Übungen.

Der Kurs „**Statistik in der Ökotoxikologie**“ behandelt die statistische Auswertung von Single-Species-Tests. Ziel ist es, das wesentliche Grundprinzip allen statistischen Testens zu vermitteln und die Teilnehmer in die Lage zu versetzen, geeignete statistische Tests auszuwählen, fachgerecht durchzuführen und deren Ergebnisse mit Sachverstand zu bewerten. Besonderes Augenmerk wird auf die Skalierung von Daten gerichtet, da die Auswahl des geeigneten Tests maßgeblich von der zugrunde liegenden Datenskala abhängt. Es werden Grundbegriffe wie Stichprobenverteilung, Seitigkeit, Irrtumswahrscheinlichkeit usw. geklärt sowie typische statistische Kenngrößen definiert. Aufbauend auf diesen Grundlagen werden ein allgemeines Schema zur Auswahl und Durchführung von statistischen Tests vorgestellt und verschiedene Tests für Biotestauswertungen besprochen (Vortests auf Ausreißer, Normalverteilung und Varianzhomogenität, Vergleiche mit einem Standard, statistische Absicherung der Wirkung zwischen zwei Testansätzen (z.B. Limit-Test, Test gegen Lösungsmittelkontrolle), allgemeiner Nachweis eines Effekts (Varianzanalyse, ANOVA), Absicherung der Wirkungsschwellenkonzentration durch multiple Tests (NOEC/LOEC), Prinzip der Bonferroni-Korrektur, ß-Fehler und Teststärke, minimum detectable difference (MDD)). Ein weiteres Schwerpunktthema ist die Modellierung von Daten (zum Beispiel Dosis-Wirkungs-Funktionen). Es wird auf die Möglichkeit der Anpassung von Funktionen eingegangen, wobei gängige Modelle für Dosis-Wirkungs-Funktionen (z.B. Probit-, Weibull- und Logit-Modell; mehrparametrige Dosis-Wirkungs-Funktionen) und die Methoden für deren Anpassung (lineare und nichtlineare Regression) vorgestellt und diskutiert werden. Die Konzepte ECx und NOEC werden miteinander verglichen und jeweilige Vor- und Nachteile erläutert. Die Theorie wird anhand von praktischen Übungen veranschaulicht und gefestigt

Als Pilotprojekt läuft derzeit der Kurs **„Environmental Exposure Assessment“.** Hier werden die Grundlagen für die mathematische Modellierung des Transports und der Transformation von Chemikalien in der Umwelt vermittelt. Ausgehend von der Massenbilanzierung in einem oder mehreren Kompartimenten werden die Konzepte zur Berechnung der Konzentrationen in Luft, Boden, Wasser und Sediment erarbeitet, in mathematische Gleichungen umgesetzt und in Übungen selbständig vertieft. Verschiedene Komplexitätsstufen vom thermodynamischen Verteilungsgleichgewicht über Fließgleichgewicht bis zum dynamischen Verhalten sowie die Aufnahme in Pflanzen und Tiere und der Transfer in Nahrungsketten werden behandelt und der Einfluss von Stoffdaten und Umweltbedingungen auf die Konzentration in den einzelnen Medien untersucht. In Fallstudien wird die Exposition (PEC = Predicted Environmental Concentration) für einzelne Chemikalien beispielhaft abgeschätzt und mit entsprechenden (Öko-) Toxizitätsdaten (PNEC = Predicted No Effect Concentration) verglichen, um das Risiko für Mensch und Ökosysteme zu ermitteln. Ansätze zur Verfeinerung der Expositionsabschätzung werden in Gruppenarbeit diskutiert und die Ergebnisse allen Teilnehmern präsentiert. Die für die Berechnungen notwendige Software wird den Teilnehmern zur Verfügung gestellt. Der Kurs wird in englischer Sprache gehalten.

**Neuer Kurs:** Weiterhin wurde in 2015 ein neuer Kurs „**Alternativmethoden**“ ins Curriculum aufgenommen. Der Kurs vermittelt folgende Inhalte: (1) ethische Grundlagen für den Schutz von Tieren als Mitgeschöpfen, (2) rechtliche Grundlagen des Tierschutzes, (3) Tierversuchsstatistiken, (4) Beispiele für Alternativen zu akuten Toxizitätstests mit Fischen (z.B. Zellkultur-Assays, Fischembryotests), (5) spezifische Endpunkte in Alternativmethoden mit Zellkulturen und Fischen, (6) Grundsätze bei der Entwicklung und Validierung von alternativen Testmethoden (z.B. ECVAM Approach, OECD-Prozedere), (7) Integration von Alternativmethoden in intelligente Teststrategien (z.B. OECD Fish Testing Strategy), (8) Non-Testing-Strategien (z.B. Waving, Read Across, QSAR-Strategien), (9) alternative Ansätze zur Bestimmung der Bioakkumulation, (10) Screening mit niederen Wirbeltieren als Alternative zu Teratogenitätstests mit Säugetieren, (11) Toxicogenomics als Alternative zu konventionellen Testmethoden. Die Module Zellkultur und Fischembryotest werden durch diverse Demonstrationen im Labor ergänzt. Den Abschluss bildet eine einstündige Klausur.

*Organisation*

Dem PGS-Gremium gehören sechs Personen an. Ein Mitglied wird jeweils aus den Fachgesellschaften entsandt und vier aus der Kursleiterschaft (siehe Tabelle für die aktuelle Besetzung). Aus ihrer Mitte werden jeweils ein Vorsitzender und ein Stellvertreter gewählt. Das PGS-Gremium entscheidet über alle Belange des Studiengangs, regelt z.B. die Prüfungsordnung und setzt Kursleiter ein. Die Geschäftsstelle des SETAC GLB am Forschungszentrum Neu-Ulrichstein, Homberg (Ohm), ist mit der Geschäftsführung des PGS beauftragt und ist u.a. für Buchungswesen, Prüfungsamt, Koordination, Finanzen und Administration der Homepage verantwortlich.

**Tabelle 2: Zuständigkeiten im Rahmen des Postgradualstudiengangs Fachökotoxikologie**

| **PGS Gremium** |  |  |
| --- | --- | --- |
| Vorsitzender | Jun.-Prof. Dr. Ralf B. Schäfer | Institut für Umweltwissenschaften, Universität Koblenz-Landau |
| Stellv. Vorsitzender | Prof. Dr.-Ing. Adolf Eisenträger | Umweltbundesamt Dessau, |
| Mitglied | Prof. Dr. Thomas Braunbeck | Aquatische Ökologie & Toxikologie, Universität Heidelberg |
| Mitglied | Prof. Dr. Jörg Oehlmann | Department Aquatic Ecotoxicology, Johann Wolfgang Goethe University Frankfurt |
| Mitglied | Prof. Dr. Andreas Schäffer | Lehrstuhl für Umweltbiologie und –chemodynamik, RWTH Aachen |
| Mitglied | Dr. Andreas Willing | BASF Personal Care & Nutrition GmbH, Düsseldorf |
| **Organisation** | SETAC GLB - Geschäftsstelle | Mesocosm GmbH am FNU Forschungszentrum Neu-Ulrichstein, Homberg (Ohm) |
| Geschäftsleiter | Prof. Dr. Klaus Peter Ebke | Institut für Gewässerschutz Mesocosm GmbH, Homberg (Ohm) |

*Die Erfolgszahlen des Postgradualstudiengangs*

Seit 2004 wurden 600 Kurse mit insgesamt über 1300 Teilnahmen angeboten. Die Teilnehmerschaft kommt zu 34 % aus Industrie, 14 % KMU, 25 % öffentlicher Dienst, rund 19 % sind Studierende und 9 % Arbeitssuchende.

450 Personen haben sich im Prüfungsamt des Studiengangs registriert und es gibt März 2016 insgesamt 450 aktive Teilnehmer, davon haben bereits 49% mehr als 4 Kurse absolviert.

24 Absolventen haben den Studiengang bereits erfolgreich abgeschlossen. Die Zeit, die die Teilnehmer zur kompletten Abwicklung benötigten, lag ca. zwischen 2 und 4 Jahren.

*Ausblick*

Die Kurse des PGS sind zurzeit weitgehend ausgebucht, sodass eine frühzeitige Anmeldung erforderlich ist.

Eine zweite Forderung des Berliner Manifestes, die Einrichtung von Masterstudiengängen Ökotoxikologie, ist mittlerweile an den Universitäten Koblenz Landau und Duisburg-Essen sowie der RWTH Aachen realisiert worden. An über 20 weiteren deutschen Universitäten werden zudem Schwerpunkte oder einzelne Veranstaltungen in der Öktoxikologie im Rahmen von verschiedenen Studiengängen angeboten. Hier hat das erfolgreiche PGS zur Fachökotoxikologin/Fachökotoxikologe (GDCh/SETAC GLB) entscheidende Impulse gegeben.

*Danksagung*

Für die Initiatoren des Postgradualstudiums war ein wesentlicher Aspekt, die Ausbildung möglichst kostengünstig anbieten zu können. Dies wird vor allem dadurch erreicht, dass die Kursleiter und Referenten ehrenamtlich tätig sind. Die Kursleiter seit Beginn (exklusive der Autoren):

Prof. Dr. T. U. Berendonk / Universität Dresden, Dr. C. Brühl / Universität Koblenz-Landau, Prof. Dr. R. Debus / Hochschule RheinMain, Prof. Dr. R. A. Düring / Universität Gießen, Prof. Dr. H. Foth / Universität Halle, Prof. Dr. H. Hollert / RWTH Aachen, Dr. D. Jungmann / Universität Dresden, Prof. Dr. M. Matthies / Universität Osnabrueck, Prof. Dr. R. Nagel / ehemals Universität Dresden, Dr. J. Römbke / ECT GmbH, Flörsheim, Prof. Dr. G. Schüürmann / UFZ-Umweltforschungszentrum Leipzig-Halle, Prof. Dr. R. Schulz / Universität Koblenz-Landau, Dr. T.B. Seiler / RWTH Aachen, Prof. Dr. A. Seitz † / ehemals Universität Mainz, Dr. F. Stock / UBA, Dessau.

Darüber hinaus unterstützte der Verband der chemischen Industrie das Projekt.

*Literatur*

Ahlers, J., Gies, A. und Wogram, J. (2004): Editorial: Studiengang Ökotoxikologie – Anforderungen aus Sicht der regulatorischen Ökotoxikologie. UWSF – Z Umweltchem Ökotox 16, 217-218.

Ahlers, J., Filser, J., Frank, H., Gies, A., Klein, W., Nagel, R. und Schüürmann,G. (2003): Ökotoxikologie soll endlich wissenschaftliches Fach werden. UWSF – Z Umweltchem Ökotox 15, 3-4.

Hollert, H., Ahlers, J., Schulz, R., Schüürmann, G., Ratte, H.T. und Nagel, R. (2005): Auf zu neuen Ufern – Postgraduale Weiterbildung mit dem zertifizierten Abschluss Fachökotoxikologin/e SETAC/GDCh beginnt 2005. UWSF – Z Umweltchem Ökotox 17 (1), 1 – 2.

Ebke K.P., Hollert H. (2007): Postgraduale Weiterbildung zur/zum Fachökotoxikologin/e GDCh / SETAC als Reaktion auf Lücken im Ausbildungssystem. UWSF – Z Umweltchem Ökotox 19, Sonderausgabe 1, 71-72.

Frische, T., Ahlers, J., Gies, A., Kussatz, C., Schulte, C. und Stolzenberg, H-C. (2007): Von der Erkenntnis zur Entscheidung – Ökotoxikologie in der regulatorischen Praxis des Umweltbundesamtes. UWSF – Z Umweltchem Ökotox 19, Sonderausgabe 1, 49-57.

Ahlers, J., Ebke, K. P. (2008): Beitragsserie Regulatorische Ökotoxikologie [Neue Herausforderungen in der regulatorischen Ökotoxikologie erfordern eine anspruchsvolle Ausbildung](http://springer.r.delivery.net/r/r?2.1.Ee.2Tp.1iOyWp.BxcYJi..N.GslC.37LE.NDGEeY00). DOI 10.1007/s12302-008-0031-3, November 15, 2008, 5-7.
